# The role of MRI in axillary lymph node imaging in breast cancer patients: a systematic review

**DOI:** 10.1007/s13244-015-0404-2

**Published:** 2015-03-24

**Authors:** V. J. L. Kuijs, M. Moossdorff, R. J. Schipper, R. G. H. Beets-Tan, E. M. Heuts, K. B. M. I. Keymeulen, M. L. Smidt, M. B. I. Lobbes

**Affiliations:** 1 Department of Radiology and Nuclear Medicine, Maastricht University Medical Center+, PO Box 5800, 6202 AZ Maastricht, The Netherlands; 2 Department of Surgery, Maastricht University Medical Center+, PO Box 5800, 6202 AZ Maastricht, The Netherlands; 3 GROW - School for Oncology and Developmental Biology, PO Box 616, 6200 MD Maastricht, The Netherlands

**Keywords:** Breast cancer, MRI, Axilla, Lymph nodes, Neoplasm metastases

## Abstract

**Objectives:**

To assess whether MRI can exclude axillary lymph node metastasis, potentially replacing sentinel lymph node biopsy (SLNB), and consequently eliminating the risk of SLNB-associated morbidity.

**Methods:**

PubMed, Cochrane, Medline and Embase databases were searched for relevant publications up to July 2014. Studies were selected based on predefined inclusion and exclusion criteria and independently assessed by two reviewers using a standardised extraction form.

**Results:**

Sixteen eligible studies were selected from 1,372 publications identified by the search. A dedicated axillary protocol [sensitivity 84.7 %, negative predictive value (NPV) 95.0 %] was superior to a standard protocol covering both the breast and axilla simultaneously (sensitivity 82.0 %, NPV 82.6 %). Dynamic, contrast-enhanced MRI had a lower median sensitivity (60.0 %) and NPV (80.0 %) compared to non-enhanced T1w/T2w sequences (88.4, 94.7 %), diffusion-weighted imaging (84.2, 90.6 %) and ultrasmall superparamagnetic iron oxide (USPIO)- enhanced T2*w sequences (83.0, 95.9 %). The most promising results seem to be achievable when using non-enhanced T1w/T2w and USPIO-enhanced T2*w sequences in combination with a dedicated axillary protocol (sensitivity 84.7 % and NPV 95.0 %).

**Conclusions:**

The diagnostic performance of some MRI protocols for excluding axillary lymph node metastases approaches the NPV needed to replace SLNB. However, current observations are based on studies with heterogeneous study designs and limited populations.

**Main Messages:**

*• Some axillary MRI protocols approach the NPV of an SLNB procedure.*

*• Dedicated axillary MRI is more accurate than protocols also covering the breast.*

*• T1w/T2w protocols combined with USPIO-enhanced sequences are the most promising sequences.*

## Introduction

Some 15 years ago, sentinel lymph node biopsy (SLNB) replaced axillary lymph node dissection (ALND) for nodal staging in clinically node-negative breast cancer patients. Although less invasive compared to ALND, the SLNB is still associated with non-neglible morbidity. For example, lymph oedema is reported in 5–8 % of patients after an SLNB [1, 2]. Other common complications are pain, paresthesia, decreased arm strength and shoulder stiffness [3]. Sixty percent of newly diagnosed breast cancer patients are pathologically node negative and consequently do not benefit from SLNB. Nonetheless, these patients are at risk of its complications.

As a result, there is a continuous search for novel, non-invasive nodal staging techniques that can accurately identify lymph node-negative breast cancer patients. Hypothetically, a non-invasive technique with high sensitivity and negative predictive value (NPV) could replace SLNB, eliminating its morbidity risk. Very small metastases will be hard to detect with any imaging technique, but recent studies have shown that these small metastases, such as isolated tumour cells (i.e., N0i+, <0.2 mm) and micrometastases (i.e., N1mi, 0.2*–*2.0 mm), do not influence overall survival. Consequently, sensitivity for detecting very small metastases is less important [4], creating a window of opportunity for (non-invasive) imaging techniques that might be able to perform axillary staging of breast cancer patients.

In recent years, many non-invasive imaging modalities, such as ultrasound or PET-CT, have been suggested for this purpose. We opted to systematically review the current evidence of axillary staging using MRI, since it appears to have several advantages over other imaging modalities, such as the lack of ionising radiation (compared to PET/CT) or less intra- and interobserver variation (common in ultrasound examinations).

The aim of this systematic review is to determine whether the diagnostic performance of MRI is sufficient to confidently exclude axillary lymph node metastasis in breast cancer patients, preventing node-negative patients from undergoing unnecessary invasive staging procedures such as SLNB.

## Materials and methods

### Search strategy

For this systematic review, the guidelines of Preferred Reporting Items for Systematic Reviews and Meta-Analyses (PRISMA) were followed [5]. A literature search was performed in the Cochrane Library, Embase and PubMed databases up to July 2014. Search terms used were breast or mamma combined with the terms neoplasms, malignancy, cancer, carcinoma and adenocarcinoma. Terms for intervention were magnetic resonance imaging, MRI, MR mammography, MR, and magnetic resonance. Terms for the reference test were axilla, axillary, lymph, node, nodal, stage, status, staging, lymph nodes, lymphatic metastases, sentinel lymph node biopsy, lymph node excision, axillary lymph node dissection, ALND, SLNB, sentinel lymph node, SN and sentinel node. Outcome terms were sensitivity, specificity, negative predictive value, NPV, positive predictive value, PPV and accuracy. A manual search of the reference lists of retrieved articles was performed for any additional publications.

### In- and exclusion criteria

To avoid selection bias, in- and exclusion criteria were established prior to the literature search. Inclusion criteria were (1) diagnostic research, (2) newly diagnosed, histologically proven breast cancer patients, (3) at least 15 patients in the final analysis, (4) patients underwent standard breast MRI or dedicated axillary MRI prior to surgery, (5) minimum magnetic field strength of 1.5 T and (6) pathological examination was based on SLNB or ALND.

Additional exclusion criteria were (1) not addressing nodal staging; (2) studies with patients undergoing any type of neo-adjuvant chemo-, immune- or endocrine therapy; (3) patients with a history of axillary surgery or treatment; (4) patients with recurrent axillary disease; (5) studies without >3 of the following diagnostic performance parameters: sensitivity, specificity, PPV, NPV or accuracy; (6) editorials, conference publications, surveys, case reports, reviews, meta-analysis, ex vivo studies and animal studies.

### Study selection

Two independent reviewers searched for eligible articles and excluded duplicates. First, irrelevant articles based on the abstract and title were excluded by one reviewer and verified by the second one. Second, predefined in- and exclusion criteria were applied. Third, the full text of the remaining articles was obtained and considered by both reviewers. The study selection process did not apply any time or language restrictions. In the last phase, only two articles were additionally excluded (Spanish and Chinese).

### Data extraction and quality assessment

Data from the included studies were extracted by both reviewers in consensus, using a standardised extraction form. The following data were collected: first author, year of publication, study design (retrospective or prospective), population size, mean patient age and range, magnetic field strength, breast MRI (i.e., covering the breast and axilla together) or ‘dedicated’ axillary MRI (i.e., specifically designed for imaging of the axilla), radiofrequency coil used, imaging sequences acquired, contrast agent used, voxel size, imaging analysis, timing of surgery, breast cancer and nodal stage at inclusion, tumour histology, pathological assessment, prevalence of nodal metastases and diagnostic performance parameters such as sensitivity, specificity, NPV, PPV and accuracy.

Both reviewers assessed the quality of the articles using the Quality Assessment of Diagnostic Accuracy Studies 2 (QUADAS-2) checklist [6]. *P*-values ≤ 0.05 were considered statistically significant. As this is a systematic review, no approval from an institutional review board was required.

## Results

**Fig. 1:**
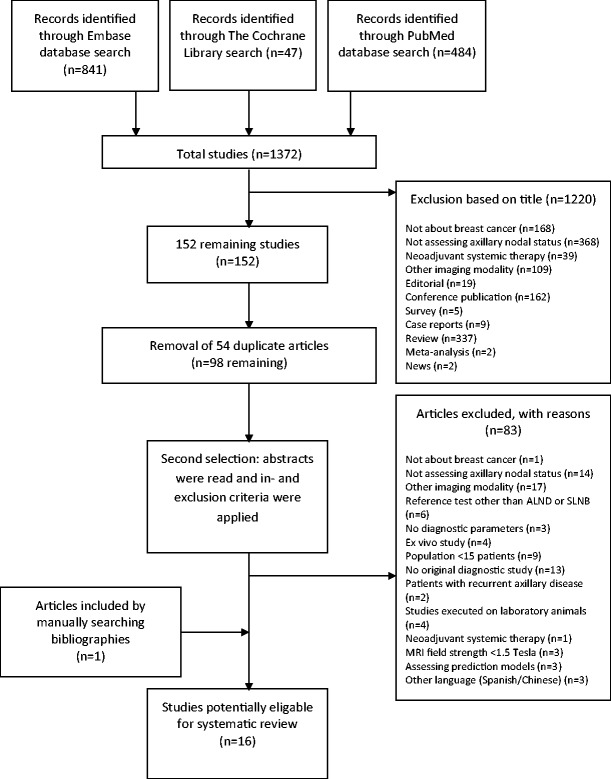
Flowchart of study selection process according to the Preferred Reporting Items for Systematic Reviews and Meta-Analyses (PRISMA)

A total of 1,372 potentially eligible studies were identified in the primary search. After a first selection 1,220 articles were excluded. Of the remaining 152 studies, duplicates between various searches were removed, leaving 98 studies. During a second selection, abstracts of the remaining 98 studies were read and in- and exclusion criteria were applied, leaving 15 studies to be reviewed. One additional study was found in the references of an included article, and after applying the in- and exclusion criteria, it was included in this review. Therefore, a total of 16 articles were selected for this systematic review [7–22]. The search and selection processes are summarised in Fig. 1.

### Publication characteristics

Twelve of the 16 included studies (75 %) had a prospective study design [9–11, 13–19, 21, 22]. The included studies contained a total of 1,591 patients (mean 99 per study, range 18–505). Patient age ranged from 43 to 62 years. Only three articles reported the exact time frame between MRI and surgery (within a mean of 6.7 days, range 1–14 days) [18, 19, 22].

### Technical MRI details

In all but one study a 1.5-T MRI was used; in the remaining study both a 1.5- and a 3.0-T MRI system was used [12]. Unenhanced T1w or T2*w imaging was used in three studies [10, 14, 16], seven studies used dynamic contrast-enhanced T1w imaging sequences [7, 9, 11, 12, 17, 20, 21], six studies used diffusion-weighted imaging (DWI) [8–10, 13, 15, 19] and two studies used ultrasmall superparamagnetic iron oxide (USPIO) enhanced T2*w imaging sequences [18, 22]. Only 5 studies used a protocol dedicated to the axilla [11, 14, 16, 18, 22]; the other 11 used a coil covering both the breast and axilla [7–10, 12, 13, 15, 17, 19–21]. Intravenous contrast administration was used in 12 studies; agents used were gadodiamide [11, 21], gadobenate dimeglumine [7–9, 20], gadoterate meglumine [19], gadobutrol [12] and USPIO ferumoxtran-10 [18, 22]. In two studies, the type of contrast was not reported[15, 17]. Criteria for distinguishing positive from negative nodes varied to a large extent; size, short/long axis ratio, lymph nodes >4 mm, short axis diameter >5 or >10 mm, shape, irregular margins, lobulated margins, presence of fatty hilum, cortical thickening, cortical thickness >3 mm, asymmetric cortex, unclear margins, perinodular oedema, matting, anatomical location of the lymph node, signal intensity time curves, heterogeneous uptake of USPIO, lack of USPIO uptake, T2* values, high signal intensity on DWI, early stage enhancement, apparent diffusion coefficient (ADC) values, visual inspection of DWI and ADC and detectability on DWI.

### Reference test

Eight studies reported the method of their pathologic analysis of the lymph nodes [7, 9, 12, 14, 16, 18, 19, 22], which consisted of sectioning of the long or short axis or both, sectioning of parallel slices of 2–4 mm thickness, H&E staining, conventional microscopic examination, the position of the lymph nodes, examination of the residual fatty tissue to detect any small lymph nodes and immunohistochemical assay. Five studies did not further specify the used pathologic examination (“histopathological examination”, “pathologically confirmed with SLNB or ALND”, “pathology was reviewed”, “analysed and examined by pathologist” and “histopathologic evaluation”) [10, 11, 13, 20, 21], and three studies did not report anything about the used analysis [8, 15, 17].

Pooling of the acquired data in meta-analyses was averted because of the very large heterogeneity of breast cancer stage and subtypes, as well as imaging techniques and pathological assessments used. Instead, descriptive statistics were used. Detailed information of the selected studies is presented in Tables 1 and 2.

**Table 1:** Study characteristics and MRI technical details

Technical MRI details									
Author	Year	Study design	Field strength	Dedicated vs. covering*	Coil	Imaging sequences	Contrast used	Voxel size	Image analysis
Yoshimura et al.	1999	P	1.5 T	Dedicated	3-Inch circular SC	T1w	None	1.3 × 0.6 × 3.0 mm3	Short and long axis ratio and morphology
Kvistad et al.	2000	P	1.5 T	Dedicated	BC and extension to axilla	T1w, dynamic T1w	Gadodiamide	2.0 × 1.0 × 4.0 mm3	Normal diameter 2–3 mm, signal intensity time curves, morphology
Michel et al.	2002	P	1.5 T	Dedicated	Cardiac SC	T1w, T2w, T2*w	Ferumoxtran-10	T1w 0.9 × 0.9 × 3.5, T2w 0.4 × 0.4 × 3.5, T2*w 0.9 × 0.9 × 2.0 mm3	Size, shape, long-shirt axis ratio, USPIO uptake patterns
Harada et al.	2007	P	1.5 T	Dedicated	Cardiac surface coil	T1w, T2*w, dynamic T2*w	Ferumoxtran-10	0.63 × 1.25 × 0.5 mm3	Pre contrast: short axis >5 or >10 mm, replacement fatty hilum, irregular margins. USPIO: heterogeneous uptake or lack of uptake
Orguc et al.	2012	P	1.5 T	Breast and axilla	Not reported	T2w, dynamic T1w	Not reported	Not reported	Size, SI time curves
Fornasa et al.	2012	P	1.5 T	Breast and axilla	4-Channel phased array coil	T1w, T2w, dynamic T1w, DWI (b-value 0/800)	Gadoterate meglumine	T2w 1.3 × 1.3 × 4.0; T1w 1.1 × 1.3 × 4.0; dynamic T1w 1.4 × 1.4 × 1.8; DWI 5.3 × 5.3 × 4.0 mm3	Lymph node area, ADC
He et al.	2012	P	1.5 T	Breast and axilla	8-Channel BC	T1w, dynamic T1w, T2w, DWI (b-value 0/500/800)	Gadopente-tate dimeglumine	Dynamic T1w 1.1 × 1.1 × 2 mm3; DWI 2.7 × 2.7 × 4 mm3; T1w not reported (only ST: 5 mm)	SI time curve, shape, margin, diameter, long/short axis ratio, node anatomical location, ADC values, high SI on DWI, early stage enhancement
Scaranelo et al.	2012	P	1.5 T	Breast and axilla	8-Channel BC	T1w, T2w, DWI (b-value 0/50/300/700/1000)	None	T1 0.7 × 0.9 × 1.2 mm3; (T2 not reported; DWI only ST: 4 mm)	Shape, presence of fatty hilum, cortex irregular, lobulated margins, visual inspection of DWI and ADC
Hwang et al.	2013	R	1.5 T	Breast and axilla	4-Channel BC	T1w, T2w, DWI (b-value 750/1000), Dynamic T1w	Gadopente-tate dimeglumine	Not reported (only ST: T1w 3.4, T2w 2.6, DWI 3.4, Dynamic T1w 2.6 mm)	Cortical thickening, irregular or round shape, loss of fatty hilum
Luo et al.	2013	P	1.5 T	Breast and axilla	8-Channel BC	T1w, T2w, DWI	None	T1w 1.0 × 0.7 × 1.0; T2w 1.1 × 1.1 × 4.03; DWI 1.0 × 0.7 × 6.0 mm3	Lymph nodes >4 mm, ADC values
Kamitani et al.	2013	R	1.5 T	Breast and axilla	Body coil	T1w, dynamic T1w, T2w, DWI			
(b-Value 0/1000)	Gadopente-tate dimeglumine	T1w 0.93 × 0.93 × 4.0 mm3; DWI 2.8 × 4.1 × 5.0 mm3 (T2w not reported)	Short axis, detectability on DWI						
Basara et al.	2013	P	1.5 T	Breast and axilla	8-Channel BC	CE-T1w, T2w, DWI			
(b-Value 0/600)	Not reported	DWI: 0.2 × 0.2 × 5.0 mm3; other sequences not reported	Size, ADC						
Hieken et al.	2013	R	1.5 T	Breast and axilla	BC	Dynamic T1w	Gadobenate dimeglumine	Not reported	Cortical thickness >3 mm, asymmetric cortex, shape, unclear margins, perinodular oedema, loss or displacement of hilum, matting
Abe et al.	2013	P	1.5 T	Breast and axilla	BC	T2w, dynamic T1w	Gadodiamide	1.0 × 1.0 mm2, ST not reported	Diffuse and asymmetrical cortical thickening, loss of hilum
Li et al.	2014	P	3.0 T	Dedicated	12-Channel body coil	T1w, T2w, T2*w	None	0.8 × 0.8 × 3.0 mm3	T2* values
An et al.	2014	R	1.5 or 3.0 T	Breast and axilla	BC	T2w, T1w, dynamic T1w	Gadobutrol	Both 1.5T and 3.0T; T2w ST 3 mm, dynamic unenhanced and CE T1w FOV 320 × 320, ST 3 mm	Loss of fatty hilum, cortex thickness of >3 mm, irregular or round shape on T2w

**Table 2:** Patient characteristics

Author	LN imaging analysis	Time to surgery (days)	*n*	Prevalence N+	Mean age in years (range)	T-stage at diag-nosis	Histologic type	Pathologic analysis
Yoshimura et al.	N-N	“Preoperative MRI”, n.o.s.	202	40.0 %	55 (30–86)	T1-T3	PT, ST, scirrhous, other	Sectioned long-axis
Kvistad et al.	P-P	“Before surgery”,n.o.s	65	37.0 %	59.4 (38–79)	T1-T4	IDC, ILC, MC, tubular, adenocarcinoma	Histopathological examination n.o.s.
Michel et al.	P-P and N-N	2 days (range 1–6 days)	18	61.1 %	53 (22–76)	T1-T4	NR	LN regarded as positive when tumour cells were present at light microscopy, independent from immunohistochemical staining results.
Harada et al.	N-N	Mean 1.2 days	33	19.0 %	58 (36–77)	T1-T4	PT, ST, scirrhous, medullary, MC, apocrien, SC, spindle cell	Sectioned long-axis, H&E staining, conventional microscopic examination
Orguc et al.	N-N	NR	155	25.8 %	43 (28–76)	NR	NR	NR
Fornasa et al.	One LN/ALND/patient	14 days	43	44.2 %	58 (39–78)	NR	IDC, ILC	Position of the LN and long × short axis
He et al.	N-N	“After MRI”, n.o.s.	79	12.0 %	44 (20–67)	NR	DCIS, IDC, ILC, lymphoma	Each LN MRI removed, residual fatty tissue examined, LN sliced 4-mm sections, 3-μm-thick slices cut from each section, stained with H&E
Scaranelo et al.	P-P	“After MRI”, n.o.s.	61	43.0 %	53 (33–78)	NR	NR	Embedded in paraffin blocks for histopathologic evaluation n.o.s.
Hwang et al.	P-P	“After MRI”, n.o.s.	349	26.4 %	51.3 (25–79)	Only T1	IDC, ILC, MC, others	Intraoperative frozen section, H&E staining. Remaining portions SLNB; sectioning and immunohistological assay
Luo et al.	N-N	“Preoperative MRI”, n.o.s.	36	57.0 %	53 (30–63)	T1-T3	DCIS, IDC, ILC, other	Analysed and examined by pathologist n.o.s.
Kamitani et al.	P-P and N-N	NR	108	23.6 %	54.9 (34–84)	Tis-T3	DCIS, IDC,ILC, MC, metaplastic	NR
Basara et al.	P-P and N-N	NR	110	24.0 %	Benign 47 (19–73); malignant 43 (29–70)	NR	IDC, ILC, IDC+ILC, IDC+MC, IDC+pleomorphic, medullary, malignant phyllodes	NR
Hieken et al.	P-P and N-N	‘Preoperative MRI”, n.o.s.	505	30.1 % with N0i+, 27.3 % without N0i+	62 (24–91)	T1-T4	IDC, ILC, mixed mammary carcinoma, other	Pathology was reviewed and the presence and extent of axillary nodal disease was verified n.o.s.
Abe et al.	P-P	NR	50	32.0 %	59.9 (33–83)	T1-T3	IDC, ILC	Pathologically confirmed with SLNB or ALND n.o.s.
Li et al.	N-N	NR	35	42.0 %	NR (30–58)	T1-T2	IDC, ILC, tubular	Parallel slices 2–3 mm thickness and stained with H&E
An et al.	P-P	‘Preoperative MRI”, n.o.s.	215	61.4 %	50 (26–83)	T1-T3	IDC, ILC, MC, invasive micro papillary carcinoma, metaplastic, medullary	Sections stained with H&E

*P-P* patient by patient*, N-N* node by node*, n.o.s.* not otherwise specified, *n* population size, *N+* positive lymph node status, *N0i +* isolated tumour cells, *IDC* invasive ductal carcinoma, *ILC* invasive lobular carcinoma, *DCIS* ductal carcinoma in situ, *PT* papillotubular*, ST* solid tubular *MC* mucinous carcinoma, *SC* squamous carcinoma*, H&E* haematoxylin and eosin, *LN* lymph node, *MRI* magnetic resonance imaging, *SLNB* sentinel lymph node biopsy, *ALND* axillary lymph node dissection, *NR* not reported

### Quality of included studies

Quality assessment of the included studies is summarised in Table 3. A significant risk of bias was observed in three included studies [9, 15, 17]. The following items scored poorly or unclear overall: the patient selection, conduct or interpretation of the index test, conduct or the interpretation of the reference standard, and flow and timing of the study.

**Table 3:**
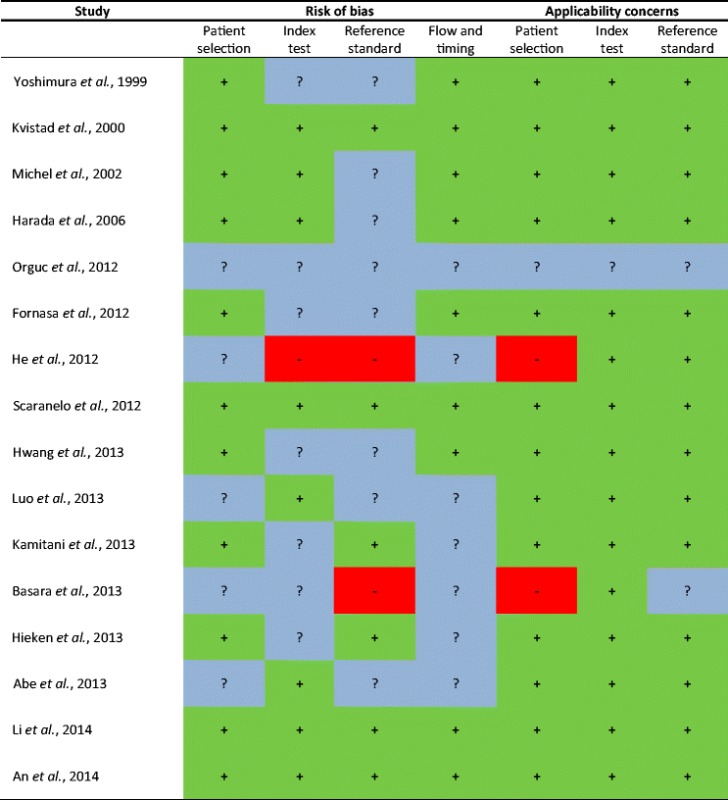
Presentation for QUADAS-2 results

Low risk,  High risk,  Unclear risk

### Diagnostic performance of MRI in axillary lymph node staging: Dedicated to the axilla versus covering the breast and axilla

Of the five studies that used a dedicated axillary coil, the three studies with T1w, T2w or dynamic contrast-enhanced (DCE) imaging sequences had a median sensitivity of 88.4 % (range 79–94.6 %) and NPV of 94.7 % (range 87–95 %) [11, 14, 16]. Two studies using T2*w imaging sequences with USPIO, had a lower median sensitivity (83.0 %; range 73.0–86.4 %), but a slightly higher NPV (95.9 %; range 78.0–98.0 %) compared to the previously mentioned three studies [18, 22]. Overall, the median sensitivity of the five studies together was 84.7 % (range 73–94.6 %) with an NPV of 95.0 % (range 78.0–98.0 %).

A coil covering both the breast and axilla was used in 11 studies [7–10, 12, 13, 15, 17, 19–21], which were divided in two groups that used different imaging sequences. For the studies using T1w, T2w or DCE, median sensitivity was 60.0 % (range 33.3–97.0 %) with an NPV of 80.0 % (range 1.9–99.5 %). For the studies using DWI, median sensitivity was 84.2 % (range 53.8–97.0 %) with an NPV of 90.6 % (range 77.8*–*99.4 %) [7–10, 12, 13, 15, 17, 19–21]. Overall sensitivity and NPV were 82.0 % (range 33.3–97.0 %) and 82.6 % (range 1.9–95.7 %), respectively. More detailed study results for articles comparing a dedicated axillary coil versus a breast and axillary coil are summarised in Table 4.

**Table 4:** Diagnostic performance of dedicated vs. standard protocol

First author, year		Sensitivity (95 % CI)	Specificity (95 % CI)	NPV (95 % CI)	PPV (95 % CI)	Accuracy (95 % CI)
Dedicated to axilla						
T1w/T2w/DCE						
Yoshimura et al. 1999		79.0 %	93.0 %	87.0 %	89.0 %	88.0 %
Kvistad et al. 2000		83.0 %	90.0 %	90.0 %	83.0 %	88.0 %
Li et al. 2014		94.6 %	98.5 %	95.0 %	98.2 %	Not reported
	*Median*	*88.4* %	*93.0* %	*94.7* %	*89.0* %	*86.5* %
	*Range*	*79–94.6* %	*90–98.5* %	*87–95* %	*83.0–98.2* %	*88.0* %
T2*w USPIO						
Michel et al. 2002	Disease based	82.0 %	100 %	78.0 %	100,0 %	89.0 %
	Two readers lymph node based	73.0*–*83.0 %	96.0*–*97.0 %	97.0*–*98.0 %	71.0*–*74.0 %	94.0*–*95.0 %
Harada et al. 2006	Combined study	86.4 %	97.5 %	96.1 %	91.1 %	95.0 %
	Postcontrast alone	84.7 %	96.8 %	95.6 %	88.5 %	94.0 %
	*Median*	*83.0* %	*97.0* %	*95.9* %	*89.8* %	*94.3* %
	*Range*	*73.0–86.4* %	*96.0–100* %	*78.0–98.0* %	*71.0–100* %	*89.0–95.0* %
	*Median*	84.7 %	96.8 %	95.0 %	89.0 %	91.5 %
	*Range*	*73–94.6* %	*90.0–100.0* %	*78.0–98.0* %	*71.0–100* %	*88.0–95.0* %
Covering breast and axilla						
T1w/T2w/DCE						
Orguc et al. 2012		89.0 %	14.0 %	80.0 %*	21.4 %*	Not reported
He et al. 2012	Overall	33.3*–*86.5 %	95.2*–*98.2 %	1.9*–*16.7 %	66.7*–*82.6 %	Not reported
	Early stage enhancement rate**	97.0 %	73.5 %	99.5 %*	30.5 %*	Not reported
Scaranelo et al. 2012		88.4 % (76–95)	82.4 % (71–90)	94.7 %*	69.4 %*	85.0 % (77–91)
Hwang et al. 2013		47.8 %	88.7 %	82.6 %	60.2 %	77.9 %
Hieken et al. 2013	N0 with N0i+	54.2 % (46.6*–*61.6)	78.2 % (73.2*–*82.5)	75.7 % (70.7*–*80.1)	57.7 % (49.9*–*65.2)	69.7 %
	N0 without N0i+	57.2 % (49.1*–*64.9)	78.2 % (73.2*–*82.5)	78.9 % (74.0*–*83.2)	56.2 % (48.2*–*63.9)	71.3 %
Abe et al. 2013		60.0 %	79.0 %	81.0 %	59.0 %	74.0 %
An et al. 2014		67.5 %	78.0 %	79.2 %	65.9 %	74.0 %
	*Median*	*60.0* %	*78.6* %	*80.0* %	*59.0* %	*74.0* %
	*Range*	*33.3–97.0* %	*14.0–98.5* %	*1.9–99.5* %	*21.4–92.6* %	*69.7–85.0* %
DWI						
Fornasa et al. 2012	Cutoff <1.09 × 10^−3^ mm^2^/s	94.7 %	91.7 %	95.7 %	90.0 %	93.0 %
He et al. 2012	Cutoff <1.35 × 10^−3^ mm^2^/s	97.0 %	54.5 %	99.4 %*	20.4 %*	Not reported
Scaranelo et al. 2012	No cutoff value	83.9 % (73–91)	77.0 % (65–86)	90.9 %*	60.5 %*	80.0 % (72–86)
Luo et al. 2013	Cutoff <0.89 × 10^−3^ mm^2^/s	82.2 %	82.4 %	77.8 %	86.1 %	82.3 %
	ADC ratio*** ≤1.097	84.4 %	88.2 %	81.1 %	90.5 %	86.1 %
Kamitani et al. 2013	Cutoff <1.05 × 10^−3^ mm^2^/s	53.8 %	86.9 %	85.9 %	56.0 %	79.1 %
	Cutoff <1.22 × 10^−3^ mm^2^/s	75.6 %	71.1 %	90.2 %	54.3 %	Not reported
Basara et al. 2013	Cutoff <1.49 × 10^−3^ mm^2^/s	95.6 %	30.3 %	95.6 %	30.3 %	Not reported
	*Median*	*84.2* %	*79.7* %	*90.6* %	*58.3* %	*82.3* %
	*Range*	*53.8–97.0* %	*30.3–91.7* %	*77.8–99.4* %	*20.4–90.5* %	*79.1–93.0* %
	*Median*	82.0 %	78.2 %	82.6 %	59.0 %	79.1 %
	*Range*	*33.3–97.0* %	*14.0–98.5* %	*1.9–95.7* %	*30.3–92.6* %	*69.7–93.0* %

*T1w* T1 weighted*, T2w* T2 weighted, T2*w T2* weighted, *N+* nodal stage positive*, N0* no regional lymph node metastasis, *N0i +* isolated tumour cells*, DCE* dynamic contrast enhanced, *DWI* diffusion-weighted imaging, T2*w T2* weighted, *USPIO* ultrasmall superparamagnetic iron oxide, *ADC* apparent diffusion coefficient, *CI* confidence interval

*Calculated parameters, **diagnostic parameter with a combination of the highest senstivity and NPV, ***ADC ratio = ratio of lymph node ADC value to the primary tumour ADC value

### Diagnostic performance of MRI in axillary lymph node staging: Studies using T1w/T2w, DCE, DWI and T2*w USPIO imaging sequences

Seven studies using dynamic, contrast-enhanced MR imaging reported a median sensitivity of 60.0 % (range 33.3–97.0 %) and NPV of 80.0 % (range 1.9–99.5 %) [7, 9, 11, 12, 17, 20, 21]. The remaining three imaging sequences reported a more comparable median sensitivity and NPV of 88.4 % (range 79.0–94.6 %) and 94.7 % (range 87.0–95.0 %) for non-enhanced T1w/T2w, 84.2 % (range 53.8–97.0 %) and 90.6 % (range 77.8–99.4 %) for DWI and 83.0 % (range 73.0–86.4 %) and 95.9 % (range 78.0–98.0 %) for T2*w USPIO [8–10, 13–16, 18, 19, 22]. More detailed results for different imaging sequences are summarised in Table 5.

**Table 5:** Results of different imaging sequences

First author, year	Prevalence N+ %		Sensitivity (95 % CI)	Specificity (95 % CI)	NPV (95 % CI)	PPV (95 % CI)	Accuracy (95 % CI)
Imaging sequences T1w/T2w							
Yoshimura et al. 1999	*40.0* %	T1w	79.0 %	93.0 %	87.0 %	89.0 %	88.0 %
Scaranelo et al. 2012	*43.0* %	T1w	88.4 % (76–95)	82.4 % (71–90)	94.7 %*	69.4 %*	85.0 % (77–91)
Li et al. 2014	*42.0* %	T2*w	94.6 %	98.5 %	95.0 %	98.2 %	Not reported
		*Median*	*88.4* %	*93.0* %	*94.7* %	*89.0* %	*86.5* %
		*Range*	*79.0–94.6* %	*82.4–98.5* %	*87.0–95.0* %	*69.4–98.2* %	*85.0–88.0* %
Imaging sequences DCE							
Kvistad et al. 2000	*37.0* %		83.0 %	90.0 %	90.0 %	83.0 %	88.0 %
Orguc et al. 2012	*25.8* %		89.0 %	14.0 %	80.0 %*	21.4 %*	Not reported
He et al. 2012	*12.0* %	Overall	33.3*–*86.5 %	95.2*–*98.2 %	1.9*–*16.7 %	66.7*–*82.6 %	Not reported
		Early stage enhancement rate**	97.0 %	73.5 %	99.5 %*	30.5 %*	Not reported
Hwang et al. 2013	*26.4* %		47.8 %	88.7 %	82.6 %	60.2 %	77.9 %
Hieken et al. 2013	*30.1* %	N0 with N0i+	54.2 % (46.6*–*61.6)	78.2 % (73.2*–*82.5)	75.7 % (70.7*–*80.1)	57.7 % (49.9*–*65.2)	69.7 %
	*27.3* %	N0 without N0i+	57.2 % (49.1*–*64.9)	78.2 % (73.2*–*82.5)	78.9 % (74.0*–*83.2)	56.2 % (48.2*–*63.9)	71.3 %
Abe et al. 2013	*32.0* %	T1w DCE	60.0 %	79.0 %	81.0 %	59.0 %	74.0 %
An et al. 2014	*61.4* %		67.5 %	78.0 %	79.2 %	65.9 %	74.0 %
		*Median*	*60.0* %	*78.2* %	*80.0* %	*59.0* %	*74.0* %
		*Range*	*33.3–97.0* %	*14.0–98.2* %	*1.9–99.5* %	*21.4–83.0* %	*69.7–88.0* %
Imaging sequences DWI							
Fornasa et al. 2012	*44.2* %	Cutoff <1.09 × 10^−3^ mm^2^/s	94.7 %	91.7 %	95.7 %	90.0 %	93.0 %
He et al. 2012	*12.0* %	Cutoff <1.35 × 10^−3^ mm^2^/s	97.0 %	54.5 %	99.4 %*	20.4 %*	Not reported
Scaranelo et al. 2012	*43.0* %	No cutoff value	83.9 % (73–91)	77.0 % (65–86)	90.9 %*	60.5 %*	80.0 % (72–86)
Luo et al. 2013	*57.0* %	Cutoff <0.89 × 10^−3^ mm^2^/s	82.2 %	82.4 %	77.8 %	86.1 %	82.3 %
		ADC ratio*** ≤1.097	84.4 %	88.2 %	81.1 %	90.5 %	86.1 %
Kamitani et al. 2013	*23.6* %	Cutoff <1.05 × 10^−3^ mm^2^/s	53.8 %	86.9 %	85.9 %	56.0 %	79.1 %
		Cutoff <1.22 × 10^−3^ mm^2^/s	75.6 %	71.1 %	90.2 %	54.3 %	Not reported
Basara et al. 2013	24.0 %	Cutoff <1.49 × 10^−3^ mm^2^/s	95.6 %	30.3 %	95.6 %	30.3 %	Not reported
		*Median*	*84.2* %	*79.7* %	*90.6* %	*58.3* %	*82.3* %
		*Range*	*53.8–97.0* %	*30.3–91.7* %	*77.8–99.4* %	*20.4–90.5* %	*79.1–93.0* %
Imaging sequences T2*w USPIO							
Michel et al. 2002	*61.1* %	Disease based	82.0 %	100 %	78.0 %	100.0 %	89.0 %
		Two readers lymph node based	73.0*–*83.0 %	96.0*–*97.0 %	97.0*–*98.0 %	71.0*–*74.0 %	94.0*–*95.0 %
Harada et al. 2006	*19.0* %	Combined study	86.4 %	97.5 %	96.1 %	91.1 %	95.0 %
		Postcontrast	84.7 %	96.8 %	95.6 %	88.5 %	94.0 %
		*Median*	*83.0* %	*97.0%*	*95.9* %	*89.8* %	*94.3* %
		*Range*	*73.0–86.4* %	*96.0–100* %	*78.0–98.0* %	*71.0–100* %	*89.0–95.0* %

*T1w* T1 weighted*, T2w* T2 weighted, T2*w T2* weighted, *N+* nodal stage positive*, N0* no regional lymph node metastasis, *N0i+* isolated tumour cells*, DCE* dynamic contrast enhanced, *CI* confidence interval. *DWI* Diffusion-weighted imaging, T2*w T2* weighted, *USPIO* ultrasmall superparamagnetic iron oxide, *ADC* apparent diffusion coefficient, *CI* confidence interval

*Calculated parameters

**Diagnostic parameter with a combination of the highest senstivity and NPV

***ADC ratio = ratio of lymph node ADC value to the primary tumour ADC value

To summarise, the most promising results seem to be achievable when using non-enhanced T1w/T2w and USPIO-enhanced T2*w sequences in combination with a dedicated axillary protocol, sensitivity 84.7 % and NPV 95.0 %.

## Discussion

Recent studies have shown that every single axillary metastasis may not require surgery. For example, Giuliano et al. (2011) showed in the ACOSOG Z011 trial that the 5-year overall survival of breast cancer patients with limited sentinel lymph node metastasis, treated with breast conservation and systemic therapy, did not decrease when omitting an ALND [92.5 %; 95 % confidence interval (CI), 90.0–95.1 %] compared to ALND (91.8; 95 % CI, 89.1–94.5 %) [23]. Furthermore, the impact of the true pathological lymph node status on adjuvant systemic treatment recommendations appears limited, thereby eliminating the need to detect every single (extremely small) metastasis [24]. These new insights have created a window of opportunity for many other non-invasive (imaging) modalities to be used in axillary lymph node staging.

In this study we systematically reviewed the available literature on MRI’s diagnostic performance for axillary nodal staging in breast cancer patients. We aimed to determine whether MRI could sufficiently exclude axillary lymph node metastasis, thereby replacing SLNB, consequently eliminating the risk of its morbidity. For this purpose, we focused on the sensitivity and NPV, as we are mainly interested in the exclusion of axillary metastases in order to omit SLNB. Therefore, the NPV should at least be non-inferior to the NPV of SLNB. A recent meta-analysis showed a false-negative rate of 8.61 % (95 % CI: 8.05*–*9.2 %) of the SLNB [25]. The calculated NPV of the SLNB in a patient group with a prevalence of 40 % of axillary metastasis equals 94.5 %. In our current observation, the most promising results seem to be achievable when using non-enhanced T1w/T2w and USPIO-enhanced T2*w sequences in combination with a dedicated axillary protocol. These protocols turned out to have a sensitivity and NPV of respectively 84.7 and 95.0 %. These results are promising, as the NPV approaches the NPV of SLNB. However, some restraint in the clinical implementation of axillary MRI should be considered because of the study limitations, such as: the heterogeneous study design, overall limited study populations, inclusion of only single-centre studies and lack of 95 % confidence intervals mentioned in studies. To implement axillary MRI in the clinical setting, these promising results should be confirmed in large, multicentre studies and various readers of the examinations. In addition, a protocol that includes non-enhanced T1w/T2w and USPIO-enhanced T2*w sequences in combination with a dedicated axillary protocol need to be considered as they seem to give the most promising results.

In our observations, a dedicated axillary protocol (for example using a surface radiofrequency coil placed on the axilla) was superior to a more standard protocol covering both the breast and axilla in the same field of view. This is only logical, as the use of surface coils and the reduced distance from the axillary to the coil improves signal-to-noise ratios, enabling the use of higher spatial resolutions. More interestingly, DWI has, despite the use of a protocol covering the breast and axilla together, an almost equal sensitivity (84.2 %), but a lower NPV (90.6 %) when compared with studies using a dedicated protocol (84.7 and 95.0 %). Unfortunately, there are no studies that investigated the use of DWI sequences in a dedicated axillary protocol.

However, the clinical implementation of DWI and USPIO-enhanced T2*-weighted imaging might be challenging. Disadvantages of the DWI are high sensitivity to motion artefacts, limited spatial resolution and more pronounced artefacts at higher field strengths. The ADC values used in diffusion-weighted imaging are dependent on scanner and *b-*values. The clinical implementation of USPIO-enhanced imaging is hampered by the necessity of administrating the contrast 24–36 h prior to MRI imaging. Moreover, UPSIO can cause side effects in up to 18 % of the examinations such as rash, pruritus, abdominal and/or lumbar pain, chest pain and an orthostatic reaction[18]. An additional disadvantage of USPIO is that it is not universally available and using USPIO for MR lymphography is ‘off-label’. These disadvantages need to be overcome, especially since the NPV approaches the NPV of the SLNB.

Given the evidence from the Z0011 trial, there may be relatively less utility for MRI evaluation of lymph nodal status because a selected number of patients would not necessarily be managed with ALND. The main gain however is that regional treatment with MRI is based on the true absence or presence of lymph node metastases compared to traditional physical examination and/or ultrasound. Axillary ultrasound was never tested for node-to-node evaluation and is not able to predict ‘true’ nodal status accurately. In combination with the Z0011 results (in which 27 % of nodal metastases remained) created reluctance among many clinicians to implement these results.

Axillary MRI evaluation with a high NPV could induce omission of the SLNB in case of negative findings, which constitute about 65 % of all breast cancer patients. Axillary MRI with high PPV could result in an SLNB in case of limited nodal metastases. Axillary MRI evaluation with high PPV could further induce two pathways when extended nodal disease is observed: axillary lymph node dissection or neoadjuvant systemic therapy and re-evaluation using MRI after completion of all chemotherapy cycles. All this would result in policy based on the true nodal burden instead of estimating it to the best of our ability using ultrasound and the clinician’s opinion regarding the Z0011 trial results.

A subgroup of patients (clinical stage T1-2 and N0 undergoing breast conservative therapy and whole-breast radiation) would not necessarily be managed with ALND: hence the utility of preoperative axillary MRI will depend on whether or not the surgeon has adopted omission of ALND in patients with minimal sentinel node disease. In order to define the importance of MRI evaluation of axillary lymph nodes as a replacement for SLNB, differentiation between minimal and more advanced nodal disease (high nodal disease burden being defined as >3 metastatic nodes in the majority of studies) must be clear. None of the selected papers compared pN status with the number of positive lymph nodes at MRI. Only He et al. mentioned a different diagnostic accuracy for nodes from level I and level III. Future studies should strongly consider the use of node-by-node analyses of the axillary lymph nodes to test whether axillary MRI can replace SLNB.

Previously, other non-invasive techniques that were considered to exclude axillary metastases were axillary physical examination (PE), axillary ultrasound (AUS) and positron emission tomography–computed tomography (PET/CT). However, these methods lack sensitivity and NPV. Sensitivity is 25–35.5 % for PE, 43.5–72.3 % for AUS and 56–62.7 % for PET/CT [12, 26]. The NPV is 81.7 % for PE, 81.6–83.3 % for AUS and 79.1 % for PET/CT [11, 12, 27–29]. Concluding, the diagnostic performance of these techniques is insufficient to exclude lymph node metastases and omit SLNB. At this point, MRI seems to be the most promising non-invasive nodal staging technique with a highest median sensitivity of 84.7 % and NPV of 95.0 %.

Based on the QUADAS-2 tool, there was an overall good quality of studies. Three studies were assessed as studies with a low methodical quality and a high risk of bias. In the study of Orguc et al. (2012), introduction of bias was unclear in all domains: the patient selection, index test, reference test, flow and timing [17]. A high risk of bias of the reference test was observed in the studies of He et al. (2012) and Basara et al. (2013). In the study of He et al. (2012), a physician who was in charge of labelling the lymph node samples took part in every MRI examination and in the surgery of every enrolled patient [9]. Basara et al. (2013) used histopathological examination as a reference test for malignant lymph nodes and clinical examination with imaging findings as reference for benign lymph nodes. Furthermore, Basara et al. (2013) and He et al. (2012) both included patients with various indications for a conventional breast MRI, not just patients with breast cancer. This increased our concerns about whether or not this study population is applicable to our research population.

Along with the more limited methodological quality of the three studies mentioned above, we noticed a great variety in the outcome of these studies compared to other (methodologically stronger) articles. Even when these three studies were excluded, the final conclusions of our review did not change.

### Study limitations

First, the included studies were very heterogeneous in their study designs. They used different imaging sequences (T1w, T2w, DCE, DWI and T2*w), different radiofrequency coils and different contrast agents (gadolinium-based and USPIO). This stopped us from pooling data and resulted in a more descriptive analysis of the results.

Second, publication and selection bias is a study limitation in every systematic review. The tendency to not publish studies with negative results might lead to an overestimation of our results. By using the PRISMA approach in combination with an extensive search, selection and inclusion, we think that the influence of publication and selection bias is limited.

## Conclusion and outlook

In summary, the diagnostic performance of MRI for assessing axillary nodal staging in breast cancer patients is promising, as the NPV approaches the NPV of the SLNB.

However, current observations are based on (single-centre) studies with heterogeneous study designs and limited populations. Thus, these finding should be interpreted with these limitations in mind.

Based on the current findings, most desirable protocol would be a dedicated axillary coil in combination with T1-weighted, T2-weighted and T2*-weighted USPIO. Additionally, DWI combined with a dedicated coil might create the opportunity to achieve even higher diagnostic values compared to the use of a protocol covering the breast and axilla. However, there are no studies that used one of these specific protocols. Future studies should consider the use of these protocols to see whether the diagnostic performance can be increased by this approach. In order to further investigate the clinical use of axillary MRI for staging of breast cancer patients, these studies should be performed in a multicentre study comprising a large number of patients, evaluated by multiple radiologists.
